# Comparison of associated features and drug treatment between co-occurring unipolar and bipolar disorders in depressed eating disorder patients

**DOI:** 10.1186/s12888-017-1243-0

**Published:** 2017-02-27

**Authors:** Mei-Chih Meg Tseng, Chin-Hao Chang, Shih-Cheng Liao, Hsi-Chung Chen

**Affiliations:** 1 Department of Psychiatry, Far Eastern Memorial Hospital, No.21, Sec. 2, Nanya S. Rd., Banciao Dist., New Taipei City, 22060 Taiwan, Republic of China; 2 Department of Psychiatry, National Taiwan University College of Medicine, Taipei, 10051 Taiwan; 3 Department of Psychiatry, National Taiwan University Hospital, Taipei, 10002 Taiwan; 4 Department of Nursing, Oriental Institute of Technology, New Taipei City, 22061 Taiwan; 5 Department of Medical Research, National Taiwan University Hospital, Taipei, 10055 Taiwan

**Keywords:** Bipolar I disorder, Bipolar II disorder, Comorbidity, Eating disorders, Hypomania, Major depression, Mania, Pharmacotherapy

## Abstract

**Background:**

To examine the differences of associated characteristics and prescription drug use between co-occurring unipolar and bipolar disorders in patients with eating disorders (EDs).

**Methods:**

Patients with EDs and major depressive episode (MDE) were recruited from psychiatric outpatient clinics. They were interviewed and completed self-administered measures assessing eating and general psychopathology. The prescribed drugs at the index outpatient visit were recorded. Clinical characteristics and prescription drugs of groups with major depressive disorder (ED-MDD), MDE with lifetime mania (ED-BP I), and MDE with lifetime hypomania (ED-BP II) were compared. Continuous variables between groups were compared using generalized linear regression with adjustments of age, gender, and ED subtype for pair-wise comparisons. Multivariate logistic regression with adjustments of age, gender, and ED subtype was employed to estimate adjusted odds ratios with 95% confidence intervals between groups.

**Results:**

Two hundred and twenty-seven patients with EDs had a current MDE. Among them, 17.2% and 24.2% experienced associated manic and hypomanic episodes, respectively. Bipolar I and II patients displayed significantly poorer weight regulation, more severe impulsivity and emotional lability, and higher rates of co-occurring alcohol use disorders than ED-MDD patients. ED-BP I patients were found to have the lowest IQ, poorest working memory, and the most severe depression, suicidality and functional impairment among all patients. Patients with ED-BP II shared affect and behavioral dysregulations with ED-BP I, but had less severe degrees of cognitive and functional impairments than ED-BP I. Patients with ED-BP I were significantly less likely than those in the ED-MDD and ED-BP II groups to be on antidepressant monotherapy, but a great rate (27%) of ED-BP I individuals taking antidepressant monotherapy had potential risk of mood switch during the course of treatment.

**Conclusions:**

Our study identified discriminative features of bipolar I and II disorders from MDD among a group of depressed ED patients. We suggest that the associated mania, hypomania, and mood lability are predictors of clinical severity and should be identified from ED patients presented with depressive features. Accurate diagnosis of bipolar disorders may have implications for pharmacotherapy in patients with EDs.

**Electronic supplementary material:**

The online version of this article (doi:10.1186/s12888-017-1243-0) contains supplementary material, which is available to authorized users.

## Background

The relationship between affective disorders and eating disorders (EDs) has been well documented [1], but much of the research focuses exclusively on major depressive disorder (MDD) and EDs. Eating disorders, having high co-morbid rates of psychiatric and physical disorders, have been conceptualized as having features overlapping with bipolar spectrum disorders [2, 3], more specifically between binge-eating related disorder and bipolar II disorder [4–6]. Studies reveal that lifetime prevalence of bipolar disorders in the ED population ranges from 0% to 34.8% in community or outpatient samples [7, 8], and 36% to 64% among inpatients [9, 10]. A few papers using diagnostic questionnaire or symptom scale have found a rate of 19% bipolar II in individuals with EDs and higher hypomania scores in individuals with binge-eating disorder (BED) than subthreshold BED [11, 12]. Several studies have investigated the correlates of EDs in patients with bipolar disorders [13–16], far fewer have examined the effects of bipolar disorder on patients with EDs [6, 8]. No studies have examined the discriminative effects of bipolar I and bipolar II disorders from MDD in patients with EDs. Recent studies adopting the Diagnostic and Statistical Manual of Mental Disorders, 5th Edition(DSM-5)’s [17] broadened criteria of EDs have further substantiated their relationship [6, 18].

Individuals were classified as having lifetime bipolar I disorder if they had ever had a manic episode, defined as a period of seven days or longer with elevated mood, and three other mania symptoms, or with irritable mood plus four other mania symptoms, with the mood disturbance resulting in marked impairment, or hospitalization. Individuals were classified as having lifetime bipolar II disorder if they had had both major depressive episodes (MDEs) and a hypomanic episode, defined by a period of four days or longer with symptom criteria similar to mania, and with an unequivocal change in function, but without a manic episode [19]. In a few other studies, concerns regarding diagnosing individuals with major depression who have a history or concomitant hypomania/mania were addressed (bipolar depression) [20–22]. Bipolar disorders are highly prevalent in clinical samples of depressed patients, with bipolar I disorder being diagnosed in 18 − 47.1% of cases, and bipolar II disorder in 7.7 − 45% [23, 24]. Compared with individuals with only major depression (unipolar depression), those with major depression and subthreshold mania/hypomania were found to have significantly higher rates of panic disorder, alcohol use disorders, and behavioral problems [25, 26]. Individuals with bipolar II disorder had even higher rates of anxiety disorders and behavioral problems than those with major depression and subthreshold mania/hypomania [26].

Bipolar II disorder is defined as a milder form of bipolar disorder with regard to hypomanic symptom intensity [17]. However, compared to individuals with bipolar I disorder, individuals with bipolar II disorder may have greater chronicity of illness and spend more time in the depressive phase of their illness [27, 28]. Some researchers have found patterns of comorbidity [15, 29], suicide attempts [14, 30], and functional impairments [31] in bipolar II disorder that are equivalent to those in bipolar I disorder, but others report higher rates of psychiatric comorbidity [30, 32] and suicide attempts [32] in bipolar II compared to bipolar I disorder. Variability in psychiatric comorbidity and functional impairments in bipolar disorders could be related to clinical heterogeneity in sample composition and index illness phase [15, 28].

The evidence of efficacy of drug treatments for EDs has been mostly weak or moderate [33]. Fluoxetine (a selective serotonin reuptake inhibitor) remains to be the only drug approved for bulimia nervosa (BN) until Lisdexamphetamine dimesylate was approved for BED by the US Food and Drug Administration in 2015 [34]. Close monitoring for emergence of hypomanic/manic symptoms is suggested when antidepressant monotherapy is utilized in the treatment of individuals with bipolar depression [35]. Differentiating bipolar I and bipolar II disorders from MDD in ED patients with clinical presentations of major depression is therefore critical for pharmacological treatment.

Based on the above reviews, we hypothesized that depressed ED patients with comorbid bipolar I and bipolar II disorders had differential features from patients with comorbid MDD and those with comorbid bipolar I and II disorders were under-treated with drugs other than antidepressants. This study aimed to examine the associated features between co-occurring bipolar and unipolar disorders in depressed ED patients. We stratify bipolar disorders into bipolar I and bipolar II considering they may have different associated features from unipolar depression. Moreover, we examined the differences of prescription drugs between the three groups of depressed ED patients in clinical practice.

## Methods

### Participants and procedures

Participants were selected from a group of ED patients (*n* = 288) who were enrolled upon their first visit to the general psychiatric clinics at a teaching hospital via a two-phase method between August 2010 and July 2014 [6]. The written informed consent was obtained from the patients before enrollment. Individuals with active psychotic symptoms, mental sub-normality, organic mental conditions, and severe medical illnesses were excluded. All study participants were ED patients with current MDE, and were diagnosed by the ED module of the Structured Clinical Interview for DSM-IV-TR Axis I disorders Patient version (SCID-I/P) [36] and Mini International Neuropsychiatric Interview (MINI) . They also underwent face-to-face interviews, assessing comorbid psychiatric diagnoses, history of self-injury behaviors or suicide attempts, and functional impairment. Diagnostic raters were one psychiatrist and two highly trained research assistants with degrees in psychology, who were monitored throughout the study to minimize rater drift. Participants underwent intelligence testing, and completed several questionnaires concerning eating pathology, affect lability, and impulsivity. The drugs prescribed at this index visit were recorded. The Institutional Review Board of National Taiwan University Hospital approved this study.

### Measures

#### SCID-I/P

An ED diagnosis in the last three months and further in the past was established using the SCID-I/P, and adapted to meet the criteria of the DSM-5. Female patients were not required to meet the criteria of amenorrhea for anorexia nervosa (AN). The minimum frequency and duration of binge-eating/purging was once per week for three months for BN and BED [17]. Participants who reported extreme weight or shape concerns, less frequent yet regular binge-eating symptoms, or binge-eating without feelings of distress, and those who did not meet other criteria for the previous disorders, were diagnosed with ED, not otherwise specified (EDNOS).

#### MINI

The MINI is a short, diagnostic interview for the DSM-IV and ICD-10 psychiatric disorders [37]. It is divided into modules, including diagnoses of major depression, bipolar disorders, anxiety disorders, and alcohol/substance use disorders. The first 40 participant interviews were audiotaped. The κ coefficients of inter-rater reliability for all diagnoses fell between .60 (social phobia) and 1.00 (major depressive episode), except that for substance use disorder (.47). We used the MINI to assess comorbid Axis I psychiatric disorders.

#### Assessment of functional impairment and suicide risks

The 87-item Structured Interview for Anorexic and Bulimic Disorders, Expert-Assessment (SIAB-EX) covers a wide range of symptoms frequently seen in EDs — body image disturbances, bulimic symptoms, social integration problems, problems associated with sexuality, depression, compulsion, and anxiety [38]. Each item was rated for present status (the last three months) and for maximum pathology in previous years (lifetime). Responses were rated on a severity scale, ranging from 0 for “symptom not present” to 4 for “symptom very much/very severely present”. Following an independent, professional translation of the Chinese version of the SIAB-EX, all items were reviewed by two psychiatrists. The assessment of functional impairment was adopted from item 53, “was your work performance objectively impaired at work or in your household?” Suicide risks were rated by item 69, “did you ever attempt to commit suicide?”, and item 70, “did you ever hurt yourself intentionally?” Severity of suicidal acts and auto-aggressive behaviors was rated according to the number of attempts or seriousness of physical injury. Inter-rater reliability (κ coefficient) for present and lifetime severity of item 53 was .76 and .74, respectively, and ranged from .53 − .78 for items 69 and 70 [39].

#### Intelligence quotient and working memory assessments

In this study, we used the short form of Wechsler Adult Intelligence Scale—Third Edition (WAIS–III) to assess the intelligence quotient in the groups. It comprises four subtests (Similarities, Matrix Reasoning, Arithmetic, and Digit Symbol substitution) and has been demonstrated to exceed other subtest combinations with respect to validity, content representation, and time saving [40]. This short form AIS-III has been used in several studies on patients with neuropsychiatric disorders [41–43]. Scores of working memory were summed by Arithmetic and Digit Span, and were converted to the estimated percentile rank according to the Taiwanese norm [44].

#### Bulimic Investigatory Test Edinburgh (BITE)

This self-reported measure includes the Symptom Scale (30 items) and the Severity Scale (6 items). The Chinese BITE has good internal consistency (Cronbach’s α = .78 and .52, respectively) and test-retest reliability (intra-class correlation reliability of .86 and .88, respectively) for the two scales [45].

#### Body Shape Questionnaire (BSQ)

The BSQ was initially developed as a 34-item questionnaire for assessing feelings about body shape, and the behavioral and emotional consequences of such feelings. The BSQ-8 used in this study, which has 8 items derived from the Chinese BSQ-34 [46] has good internal consistency (Cronbach’s alpha, α = .88).

#### Affect Lability Scale (ALS)

This study adopted an 18-item short form (ALS-18) that was established by selecting items from the original 54-item ALS using exploratory factor analysis with eigenvalues greater than 1 [47]. The original ALS assesses people’s endorsements regarding the tendency of their mood to shift between what they consider normal, to the affective domains of anger, depression, elation, and anxiety, as well as their tendency to oscillate between depression and elation, and between depression and anxiety [48]. The reliability test showed excellent internal consistency for the Chinese version of the ALS-18 (Cronbach’s α = .93) and item-total correlations ranged from .37 to .76.

#### Beck Depression Inventory (BDI)

The BDI is a 21-item measure examining depressive symptom severity over the last week. Each question has at least four response options of differing intensities. A value ranging 0–3 is assigned to each answer and the total score determines depression severity. The Chinese version of the BDI has shown good reliability and construct validity [49]. and showed good internal consistency in this study (Cronbach’s α = .91).

#### Barratt Impulsiveness Scale (BIS)

This is a 30-item self-report questionnaire measuring trait impulsivity [50]. The Chinese version of BIS-11 (BIS-11-CH) is modified from the original BIS by removing five items (item 15, 21, 23, 27, and 29) with poor item-total correlations (less than .1) [51]. The remaining 25 items of the BIS-11-CH had a Cronbach’s α of .83, and an intact factorial structure of inability to plan (factor 1), lack of perseverance and self-control (factor 2), and propensity toward novelty-seeking and acting without thinking (factor 3) [51].

### Statistical analysis

Clinical and demographic characteristics of groups with MDD (ED-MDD), MDE with lifetime hypomanic episodes (ED-BP II), and MDE with lifetime manic episodes (ED-BP I) were compared. Descriptive statistics were computed to determine means and standard deviations of all continuous variables and frequency distributions for categorical variables. The differences of demographic data between groups were compared using ANOVA and Chi-square tests for continuous variables and categorical variables, respectively. With apparent differences in the distribution of gender and ED subtypes in different comorbid groups, multivariate analyses were therefore applied. Continuous variables between groups were compared using generalized linear regression with adjustments of age, gender, and ED subtype for pair-wise comparisons. Multivariate logistic regression with adjustments of age, gender, and ED subtype was employed to estimate adjusted odds ratios (AORs) with 95% confidence intervals (CIs) between groups. Since there were four subtypes in ED patients, three dummy variables were involved in the regression model. All tests were two-tailed, and *p* < .05 was considered significant. All statistical analyses were performed using SAS, version 9.4 (Cary, North Carolina, USA).

## Results

### Demographics and clinical characteristics

Among the ED patients, 227 were diagnosed to have a current MDE. Patients who had current MDE had a later age of onset of ED compared to those who did not have (20.1 ± 6.3 vs. 18.1 ± 4.3). There were no differences of age, gender, and educational levels between the two groups. Table 1 shows the comparison of demographics and distribution of ED subtypes between ED-MDD (58.6%), ED-BP II (24.2%), and ED-BP I (17.2%). No statistically significant differences in age, age at ED onset, and age at depression onset, were observed between groups. Patients with ED-BP I had the lowest educational level among all. The diagnostic distribution of AN, BN, BED, and EDNOS was 41 (18.1%), 101 (44.5%), 58 (25.6%), and 27 (11.9%), respectively. ED-BP I and ED-BP II comprised of higher percentages of BN and BED patients. There were statistically significant differences of sex distribution, age, age of onset of disordered eating, and BMI among ED subtypes (Additional file 1: Table S1.1). There was less female predominance in BED group compared to AN and BN groups. Age differences only existed between BED and BN patients that patients with BED were significantly older and had a later age of onset of eating problems than patients with BN. Patients with BED were the heaviest among all for both current and previous body weight. No statistically significant difference in educational level was observed among ED subgroups. However, other EDs had significantly higher risks of association with generalized anxiety disorder, social phobia, panic disorders, agoraphobia and alcohol use disorder than AN but showed comparable risks of association with most psychiatric disorders between themselves (Additional file 1: Table S1.2).

**Table 1:** Comparison of demographics and clinical variables for eating disorder patients during major depressive episodes

Variables	Eating disorders with MDD (*n* = 133)	Eating disorders with bipolar II disorder (*n* = 55)	Eating disorders with bipolar I disorder (*n* = 39)	*P*-value
Gender, female (N, %)	124 (93.2)	46 (83.6)	31 (79.5)	**0.0254**
Age (years)	27.3 ± 7.4	28.2 ± 7.2	28.4 ± 8.8	0.6184
Age at onset of disordered eating (years)	20.3 ± 6.3	19.2 ± 4.4	20.4 ± 8.1	0.5211
Age at onset of depression (years)	22.8 ± 6.9	22.2 ± 6.7	21.4 ± 8.0	0.6032
Education (years)	14.4 ± 2.3	15.0 ± 2.4	13.0 ± 2.7	**0.0005**
Eating disorder subtypes (N, %)				**0.0465**
Anorexia Nervosa	32 (24.1)	4 (7.3)	5 (12.8)	
Bulimia Nervosa	60 (45.1)	25 (45.5)	16 (41.0)	
Binge eating Disorders	25 (18.8)	19 (34.6)	14 (35.9)	
Eating disorders NOS	16 (12.0)	7 (12.7)	4 (10.3)	

Values in bold type indicated statistically significant

*MDD* major depressive disorder

Values indicate N (%) or Mean ± SD

### Severity of psychopathology and psychiatric comorbidity

Information concerning clinical correlates of the ED with different affective disorders adjusting with sex, age, and ED subtypes is presented in Tables 2 and 3. Patients with ED-BP I had the lowest intelligence quotient and working memory score among all. Compared to the ED-MDD group, those with ED-BP I/BP II had statistically significantly severe degrees of mood lability and impulsiveness, higher current body weight and binge-eating/purging severity. Moreover, patients with ED-BP I/BP II also had a significantly higher rate of comorbidity with alcohol use disorders than ED-MDD patients. Patients with ED-BP I also had a two-fold higher co-occurrence rate of panic disorder than patients with ED-MDD. In comparison to ED-MDD and ED-BP II patients, patients with ED-BP I had more severe depression, mood lability and a greater rate of severe role impairments. The proportion of patients with ED-BP II who had attempted suicide (29%) fell between that of the bipolar I and MDD groups (36% and 17%, respectively). Only the difference between the latter two groups was statistically significant. Considering AN had distinct features of psychiatric co-morbidities from other EDs, we re-analyzed the data after excluding patients with AN. Significant differences of affective lability and current body weight between ED-MDD and ED-BP II group no longer existed and there were no significant difference of percentage of suicidal acts between groups (Additional file 2: Table S2.1 − 2.4). We also provide Additional file 3: Table S3.1 − 3.3 to show the comparison of psychiatric co-morbidity in different ED subtype patients with three groups of affective disorders. Of note, small sample size in some co-morbidity categories may cause difficulties or instability in estimation of statistical significance.

**Table 2:** Comparison of psychological assessments and BMI among depressed eating disorder patients with or without lifetime hypomania/mania episodes

Variables	Eating disorders with MDD	Eating disorders with bipolar II disorder	Eating disorders with bipolar I disorder	2 vs. 1	3 vs. 1	3 vs. 2
	(1)	(2)	(3)			
	(*n* = 133)	(*n* = 55)	(*n* = 39)			
	Mean ± SD	Mean ± SD	Mean ± SD	*P*-value^†^	*P*-value^†^	*P*-value^†^
Intelligence quotient	101.2 ± 13.7	100.9 ± 12.7	93.0 ± 14.6	0.6897	**0.0085**	**0.0070**
Working memory	54.9 ± 26.5	54.6 ± 29.2	36.4 ± 27.7	0.8742	**0.0003**	**0.0026**
Bulimic Investigatory Test Edinburgh	29.7 ± 11.6	31.8 ± 9.1	30.4 ± 11.7	0.0695	0.3315	0.4221
Symptom subscale	20.4 ± 6.4	21.3 ± 5.4	19.4 ± 6.3	0.2238	0.5335	0.0760
Severity subscale	9.3 ± 6.6	10.5 ± 5.1	10.9 ± 7.3	0.0612	**0.0334**	0.7174
Body Shape Questionnaire-8	4.6 ± 1.2	4.9 ± 1.1	4.4 ± 1.3	0.0870	0.5152	**0.0465**
Beck Depression Inventory	26.2 ± 10.6	26.8 ± 8.8	31.7 ± 9.5	0.9275	**0.0079**	**0.0125**
Affective Lability Scale	48.3 ± 8.7	51.6 ± 7.9	56.7 ± 8.6	**0.0410**	**<0.0001**	**0.0039**
Barrett Impulsiveness Scale	58.5 ± 6.0	61.6 ± 6.9	62.4 ± 6.9	**0.0018**	**0.0006**	0.5505
BMI, current (kg/m^2^)	20.3 ± 4.8	23.5 ± 5.5	23.6 ± 6.2	**0.0488**	**0.0430**	0.8204
BMI, maximal (kg/m^2^)	23.8 ± 5.1	26.8 ± 6.4	25.7 ± 5.4	**0.0243**	0.4736	0.3549
BMI, minimal (kg/m^2^)	17.0 ± 3.2	18.7 ± 2.9	18.5 ± 3.8	0.2187	0.3413	0.8838

Values in bold type indicated statistically significant

*MDD* major depressive disorder, *BMI* Body mass index

^†^adjusted for age, gender, and eating disorder subtypes

**Table 3:** Comparison of lifetime psychiatric diagnoses among depressed eating disorder patients with or without lifetime hypomania/mania episode

Variables	Eating disorders with MDD	Eating disorders with bipolar II disorder	Eating disorders with bipolar I disorder	2 vs. 1	3 vs. 1	3 vs. 2
	(1)	(2)	(3)			
	(*n* = 133)	(*n* = 55)	(*n* = 39)			
	N (%)	N (%)	N (%)	AOR (95% CI)	AOR (95% CI)	AOR (95% CI)
Comorbid diagnosis						
Generalized anxiety disorder	50 (37.6)	27 (49.1)	20 (51.3)	1.31 (0.68, 2.54)	1.50 (0.71, 3.17)	1.17 (0.49, 2.79)
Social phobia	46 (34.6)	23 (41.8)	18 (46.2)	1.29 (0.65, 2.55)	1.63 (0.76, 3.50)	1.21 (0.52, 2.82)
Panic disorder	16 (12.0)	14 (25.5)	12 (30.8)	2.02 (0.89, 4.60)	**2.73 (1.12, 6.69)**	1.47 (0.56, 3.80)
Agoraphobia	27 (20.3)	13 (23.6)	15 (38.5)	0.97 (0.44, 2.11)	2.14(0.95, 4.83)	2.25 (0.87, 5.83)
Obsessive compulsive disorder	38 (28.6)	19 (34.6)	17 (43.6)	1.23 (0.61, 2.47)	1.80 (0.84, 3.85)	1.52 (0.64, 3.60)
Post-traumatic stress disorder	15 (11.3)	22 (20.0)	5 (12.8)	1.79 (0.74, 4.33)	1.13 (0.37, 3.44)	0.59 (0.18, 1.89)
Alcohol abuse/dependence	5 (3.8)	12 (21.8)	12 (30.8)	**7.68 (2.41, 24.44)**	**14.55 (4.37, 48.41)**	2.05 (0.74, 5.70)
Drug abuse/dependence	23 (17.3)	10 (18.2)	10 (25.6)	1.15 (0.49, 2.69)	1.67 (0.69, 4.06)	1.48 (0.53, 4.09)
Functional impairment^b^				0.87 (0.45, 1.68)^a^	**4.46 (1.80, 11.04)** ^a^	**5.11 (1.91, 13.72)** ^a^
Severe and very severe	67 (50.4)	26 (47.3)	32 (82.1)			
Marked	45 (33.8)	18 (32.7)	7 (18.0)			
None and mild	21 (15.8)	11 (20.0)	0			
Suicide acts^c^	23 (17.3)	16 (29.1)	14 (35.9)	1.72 (0.80, 3.70)	**2.52 (1.10, 5.77)**	1.39 (0.57, 3.40)
Auto-aggressive behaviors^d^	41 (30.8)	15 (27.3)	18 (46.2)	0.81 (0.39, 1.67)	1.92 (0.89, 4.11)	2.18 (0.89, 5.37)

AOR: odd ratio adjusted for age, gender, and eating disorder subtypes

Values in bold type indicated statistically significant

*MDD* major depressive disorder

Impairments and suicide were assessed using the Structured Interview on Anorexic and Bulimic Disorder, Expert-Assessment (SIAB-EX)

^a^Patients with severe and very severe impairments compared with all others

^b^: rated by the Item 53 ‘Was your work performance objectively impaired at work or in your household ?”. Severity of impairment is rated from 0 (=no) to 4 (=very severe)

^c^: rated by the item 69 ‘Did you ever attempt to commit suicide?’

^d^: rated by the Item 70 ‘Did you ever hurt yourself intentionally?’. Severity of suicidal acts and auto-aggressive behaviors is rated from 0 (= symptom not present) to 4 (= symptom very much/very severely present) according to the number of attempts or seriousness of physical injury; here suicide acts indicated the severity more than one serious attempt or many minor attempts and auto-aggressive behaviors indicated the severity more than marked degree

**Table 4:** Comparison of pharmacotherapy among depressed eating disorder patients with or without lifetime hypomania/mania episode at enrollment

Variables	Eating disorders with MDD	Eating disorders with bipolar II disorder	Eating disorders with bipolar I disorder	2 vs. 1	3 vs. 1	3 vs. 2
	(1)	(2)	(3)			
	(*n* = 133)	(*n* = 55)	(*n* = 39)			
	N (%)	N (%)	N (%)	AOR (95% CI)	AOR (95% CI)	AOR (95% CI)
Antidepressant monotherapy	61 (48.0)	31 (56.4)	10 (27.0)	1.23 (0.62, 2.42)	**0.35 (0.15, 0.83)**	**0.28 (0.11, 0.72)**
mood stabilizer monotherapy	0	1 (1.8)	0	NA	NA	NA
Atypical antipsychotic monotherapy	32 (25.2)	6 (10.9)	9 (24.3)	0.41 (0.15, 1.09)	1.04 (0.41, 2.61)	2.65 (0.82, 8.59)
Antidepressant plus mood stabilizers	1 (0.8)	0	1 (2.7)	NA	NA	NA
Antidepressant plus antipsychotic	17 (13.4)	10 (18.2)	9 (24.3)	1.44 (0.56, 3.47)	2.09 (0.81, 5.37)	1.36 (0.46, 4.03)
Antidepressant plus mood stabilizers and antipsychotics	1 (0.8)	1 (1.8)	1 (2.7)	2.26 (0.13, 39.12)	4.00 (0.23, 68.78)	1.78 (0.10, 30.80)
Any antidepressant	80 (63.0)	42 (76.4)	21 (56.8)	1.70 (0.78, 3.72)	0.70 (0.31, 1.60)	**0.38 (0.15, 0.99)**
Any mood stabilizer	5 (3.9)	3 (5.5)	6 (16.2)	1.01 (0.25, 4.88)	**3.95 (1.06, 14.71)**	3.96 (0.90, 17.44)
Any atypical antipsychotic	51 (40.2)	17 (30.9)	21 (56.8)	0.72 (0.36, 1.47)	2.13 (0.97, 4.65)	**2.92 (1.18, 7.20)**

*MDD* major depressive disorder

Data mission for 6 and 2 persons in eating disorders with MDD and eating disorders with bipolar I disorder, respectively

AOR: odd ratio adjusted for age, gender, and eating disorder subtypes

Values in bold type indicated statistically significant

### Pharmacotherapy among different groups

Antidepressants were the most frequently prescribed medication (63.0%), either as monotherapy (44.9%) or concomitantly with other drugs, for ED patients with a current MDE (Table 4). The percentage of patients prescribed antidepressant monotherapy in ED-BP I and ED-BP II was statistically significantly different from ED-MDD groups. Although ED-BP I patients were significantly less likely than those in the ED-MDD (AOR = 0.35, 95% CI = 0.15 − 0.83, *p* = .017) and ED-BP II (AOR = 0.28, 95% CI = 0.11 − 0.72, *p* = .008) groups to be on antidepressant monotherapy, a great rate of ED-BP I individuals taking antidepressant monotherapy (27%) was exposed to the risk of mood switch during the course of treatment. The second most commonly prescribed pharmacotherapy was atypical antipsychotic monotherapy (20.7%), followed by combination therapy of antidepressants and atypical antipsychotics (15.9%). Patients with ED-BP I disorder were more likely to be taking atypical antipsychotics than those with ED-BP II disorder, and were also more likely to be taking mood stabilizers and less likely to be taking antidepressants than those with ED-MDD. No differences presented in other comparisons between groups.

## Discussion

Using structured interviews and self-administered questionnaires, we found that lifetime hypomania and mania were prevalent in ED patients with a current MDE, with bipolar I disorder being diagnosed in 17.2% and bipolar II in 24.2% of patients. There were varying associated psychopathology across groups with different comorbidities. ED-BP I and ED-BP II patients tended to have a greater body weight and increased suicidality, greater affect lability, impulsiveness, and a higher rate of alcohol use disorder than ED-MDD patients. Patients with ED-BP I reported the most severe binge-eating, depression, affect lability, impaired intelligence and working memory, and lifetime functional impairments. Although patients with ED-BP I had statistically significantly severe degrees of impulsiveness and higher co-occurrence rates of panic disorder and alcohol use disorder than patients with ED-MDD, they did not differ from patients with ED-BP II in these dimensions. Twenty-seven percent of ED-BP I patients were treated with antidepressant monotherapy that left this subgroup of patients unprotected from future mood switch and increased likelihood of chronicity of illness.

This study successfully illustrated heterogeneity among ED patients with a current MDE. Anxiety, impulsivity, suicide attempts, and alcohol/drug use disorders are prevailing comorbidities observed in ED patients [5, 52–54]. Our study found that ED-BP I patients had a two-fold higher rate of comorbidity with panic disorder and ED-BP I and ED-BP II patients had a higher rate of comorbidity with alcohol use disorders (14- and 7-fold, respectively) compared to those with ED-MDD. This observation was consistent with prior findings that individuals with bipolar spectrum disorders had significantly higher rates of many psychiatric disorders, including panic disorder and alcohol use disorders [31]. It is noteworthy that prior suicide attempts were reported by approximately 30% of our ED patients with bipolar I and bipolar II disorders and that the suicide risk was two-fold higher in this population compared to ED patients with comorbid MDD. These findings were in line with two earlier studies reporting that suicidal or self-destructive behavior in ED patients is associated with bipolar disorder [55, 56]. The presence of EDs has been implicated as a marker of clinical severity in patients with bipolar disorders, including more frequent suicide attempts than among those without EDs [13, 14, 57]. The co-existence of EDs and bipolar disorders, especially bipolar I, indicates greater risks of suicide and psychiatric comorbidity in both groups of patients.

It is interesting to notice that heterogeneity also existed between patients with ED-BP I and ED-BP II when compared to those with ED-MDD. Patients with ED-BP II appeared to share affect and behavioral dysregulations with ED-BP I, but did not have as severe degrees of cognitive and functional impairments as ED-BP I. This finding added more evidence to support earlier concerns that failure to recognize the history or co-existence of hypomania symptoms in patients with MDE might result in no or correspondingly inadequate treatment as discussed as follows.

Although the benefits of antidepressant monotherapy remain unclear in the treatment of bipolar depression [35], it is the most common initial prescription in all ED patients with a current MDE. Patients with ED-bipolar II had a comparable rate of being prescribed antidepressant monotherapy with patients with ED-MDD, and were more likely currently taking antidepressants monotherapy than patients with ED-bipolar I, leaving the concern that patients with hypomania might be less likely than those with mania to be identified from patients with MDEs or they had received adequate treatment for associated problems by taking antidepressants monotherapy. Antidepressant monotherapy may, however, expose ED patients with comorbid bipolar spectrum disorders to a potentially increased risk of mood lability and poor impulse control, among other dangerous consequences. There is a growing consensus from experts that antidepressant monotherapy should be avoided in patients with bipolar I disorder, patients with bipolar depression with two or more concomitant manic symptoms, and in bipolar patients with high mood instability or with a history of rapid cycling [35, 58]. Several researchers have addressed the role of affect lability or hypomania symptoms in binge-eating behaviors or other impulsive behaviors in ED patients [12, 59]. With comparison to the recognition of history of mania, hypomania, or mixed episodes, mood lability may be a more obvious indicator to avoid antidepressant monotherapy.

Several open series studies [60] or double-blind placebo-controlled studies [61–63] have demonstrated that atypical antipsychotics, e.g. olanzapine and quetiapine, have efficacy in reducing depressive or obsessive symptoms and causing weight gain in patients with AN. However, studies examining the efficacy of atypical antipsychotics in reducing binge-eating in patients with BN are scarce. Use of atypical antipsychotics in the treatment of EDs was not uncommon among our patients (40.2% − 56.8%). Although meta-analysis studies have yielded disappointing result [64], it is believed that some ED patients might benefit from antipsychotic medication [33, 65, 66]. Mood stabilizers have not had a large role in the treatment of EDs and topiramate (an anticonvulsant) had well-established efficacy in reducing binge-eating frequency and weight loss [67]. Both were not used frequently in our sample. Our results provide support for the rationale of the use of antipsychotics or mood stabilizers in the treatment of ED patients if comorbid bipolar disorders can be identified via the recognition of associated features reported in this present study. We hypothesize that treatment effects on affect lability and impulsivity might offset adverse effects of weight gain by taking antipsychotics or mood stabilizers and are beneficial for eating and weight control in a subgroup of ED patients with co-occurring bipolar disorders. However, further work to determine the indication and efficacy of atypical antipsychotics or mood stabilizers in the treatment of EDs is merited.

This study did have a few methodological limitations. The first was the small sample size. Consequently, all types of EDs were grouped together. Different ED subtypes may have different risks of association with psychiatric morbidities that would have influences on our current findings. Second, participants were selected in a tertiary center in a non-Western country, and thus, the sample may not be representative of the population with EDs elsewhere. Third, the medication use reported in this study was per psychiatrists’ prescriptions from their initial encounters with patients. We did not follow up any changes in prescription at subsequent visits, or check patients’ compliance in taking these medications. Finally, this is a cross-sectional design, and diagnosis of lifetime hypomania/mania was per patients’ self-reports. Recall bias may exist. In spite of these limitations, to our knowledge, this is a pioneering work, examining the influence of bipolar disorders on ED patients during MDEs. It has important implications for pharmacotherapy in the management of ED.

## Conclusions

Our study identified discriminative features of bipolar I and II disorders from MDD among a group of depressed ED patients. It is critical to recognize the presence of mood lability, a history of hypomanic/manic episodes, and other features associated with bipolar disorders, to evaluate if individuals with major depression may have hidden bipolarity. The co-occurrence of bipolar disorders in ED patients may provide clues indicating disease severity and the likelihood of presentation of impulsiveness, suicidality, psychiatric comorbidities, and even cognitive and functional impairments. The use of antidepressants in patients with EDs should be considered carefully if they have co-existent dysregulated mood and behavioral symptoms.

## Additional files

Additional file 1: Table S 1.1. Comparison of demographics and clinical variables among ED subtypes. **Table S 1.2.** Comparison of lifetime psychiatric diagnoses between ED subtypes. (DOCX 32 kb)
Additional file 2: Table S 2.1. Comparison of demographics and clinical variables for eating disorder patients (excluding AN) during major depressive episodes. **Table S2.2.** Comparison of psychological assessments and BMI among depressed eating disorder patients (excluding AN) with or without lifetime hypomania/mania episodes. **Table S2.3.** Comparison of lifetime psychiatric diagnoses among depressed eating disorder patients (excluding AN) with or without lifetime hypomania/mania episode. **Table S2.4** Comparison of pharmacotherapy among depressed eating disorder patients (excluding AN) with or without lifetime hypomania/mania episode at enrollment. (DOCX 42 kb)
Additional file 3: Table S 3.1. Comparison of lifetime psychiatric diagnoses among depressed anorexia nervosa patients with or without lifetime hypomania/mania episode. **Table S 3.2.** Comparison of lifetime psychiatric diagnoses among depressed bulimia nervosa patients with or without lifetime hypomania/mania episode. **Table S 3.3.** Comparison of lifetime psychiatric diagnoses among depressed binge eating disorder patients with or without lifetime hypomania/mania episode. (DOCX 39 kb)
