# Supplementary material for: Comparison of associated features and drug treatment between co-occurring unipolar and bipolar disorders in depressed eating disorder patients
Source: BMC Psychiatry. 2017 Feb 27;17:81. doi: 10.1186/s12888-017-1243-0 (PMC5327542; doi:10.1186/s12888-017-1243-0)
Supplement: Additional file 1: Table S 1.1. — Comparison of demographics and clinical variables among ED subtypes. Table S 1.2. Comparison of lifetime psychiatric diagnoses between ED subtypes. (DOCX 32 kb) [file 12888_2017_1243_MOESM1_ESM.docx]

Table S 1.1 Comparison of demographics and clinical variables among ED subtypes

|  | AN (1)  (*n* = 41) | | BN (2)  (*n* = 101) | | BED (3)  (*n* = 58) | | EDNOS (9)  (*n* = 27) | | ***P v*alue** | **Post hoc**  **Comparison** |
| --- | --- | --- | --- | --- | --- | --- | --- | --- | --- | --- |
| Gender, female (N, %) | 39 (95.1) | | 93 (92.1) | | 46 (79.3) | | 23 (85.2) | | .048^†^ | 1&3^*^, 2&3^*^ |
| Age (yrs) | 27.3 | 8.6 | 25.9 | 6.9 | 29.9 | 7.8 | 29.8 | 6.6 | .004 | 3 > 2 |
| Age at onset of disordered eating | 20.6 | 6.9 | 18.2 | 4.1 | 22.9 | 8.2 | 20.0 | 4.9 | < .001 | 3 > 2 |
| Age at onset of depression | 22.2 | 5.7 | 21.3 | 6.3 | 24.3 | 8.2 | 23.1 | 7.7 | .076 | ⎯ |
| Education (yrs) | 14.2 | 2.3 | 14.4 | 2.5 | 14.3 | 2.5 | 14.1 | 2.4 | .913 | ⎯ |
| BMI, current (kg/m^2^) | 15.5 | 2.7 | 21.6 | 3.0 | 26.2 | 6.1 | 21.3 | 4.8 | < .001 | 3 > 2, 9 > 1 |
| BMI, maximal (kg/m^2^) | 22.5 | 5.7 | 24.5 | 4.8 | 27.4 | 6.1 | 24.3 | 5.3 | < .001 | 3 > 1, 2 |
| BMI, minimal (kg/m^2^) | 13.5 | 2.5 | 18.1 | 2.2 | 19.7 | 3.8 | 17.9 | 3.0 | < .001 | 3 > 2, 9 > 1 |

AN: anorexia nervosa; BN: bulimia nervosa; BED: binge-eating disorder; EDNOS: eating disorders, not otherwise specified; BMI: Body mass index;

* The percentage of female gender was significantly different between BED and AN/BN.

Values are N (%) or mean ± SD.

^†^ Fisher’s exact test

Table S 1.2 Comparison of lifetime psychiatric diagnoses between ED subtypes

| Comorbid diagnosis | AN  (1)  (*n* = 41) | BN  (2)  (*n* =101) | BED  (3)  (*n* = 58) | EDNOS  (9)  (*n* = 27) | 2 vs.1 | 3 vs.1 | 9 vs. 1 | 3 vs.2 | 9 vs. 2 | 9 vs. 3 |
| --- | --- | --- | --- | --- | --- | --- | --- | --- | --- | --- |
|  | N (%) | N (%) | N (%) | N (%) | AOR (95% C.I.) | AOR (95% C.I.) | AOR (95% C.I.) | AOR (95% C.I.) | AOR (95% C.I.) | AOR (95% C.I.) |
| GAD | 9 (22.0) | 45 (44.6) | 32 (55.2) | 11 (40.7) | **2.9** (1.2, 6.6) | **4.4** (1.8, 10.8) | 2.4 (0.8, 7.1) | 1.5 (0.8, 2.9) | 0.9 (0.4, 2.0) | 0.6 (0.2, 1.4) |
| SOP | 10 (24.4) | 42 (41.6) | 20 (34.5) | 15 (55.6) | 2.2 (1.0, 5.0) | 1.6 (0.7, 4.0) | **3.9** (1.4, 11.0) | 0.7 (0.4, 1.4) | 1.8 (0.7, 4.1) | 2.4 (0.9, 6.0) |
| PD | 2 (4.9) | 19 (18.8) | 15 (25.9) | 6 (22.2) | 4.5 (1.0, 20.4) | **6.8** (1.5, 31.7) | 5.6 (1.0, 30.1) | 1.5 (0.7, 3.3) | 1.2 (0.4, 3.5) | 0.8 (0.3, 2.4) |
| AGO | 2 (4.9) | 28 (27.7) | 16 (27.6) | 9 (33.3) | **7.5** (1.7, 33.1) | **7.4** (1.6, 34.4) | **9.8** (1.9, 49.8) | 1.0 (0.5, 2.0) | 1.3 (0.5, 3.2) | 1.3 (0.5, 3.5) |
| OCD | 11 (26.8) | 35 (34.7) | 19 (32.8) | 9 (33.3) | 1.4 (0.6, 3.2) | 1.3 (0.6, 3.2) | 1.4 (0.5, 3.9) | 0.9 (0.5, 1.8) | 0.9 (0.4, 2.3) | 1.0 (0.4, 2.7) |
| PTSD | 1 (2.4) | 17 (16.8) | 9 (15.5) | 4 (14.8) | 8.1 (1.0, 63.0) | 7.3 (0.9, 60.5) | 7.0 (0.7, 66.0) | 0.9 (0.4, 2.2) | 0.9 (0.3, 2.8) | 0.9 (0.3, 3.4) |
| AUD | 2 (4.9) | 12 (11.9) | 7 (12.1) | 8 (29.6) | 2.6 (0.6, 12.3) | 2.7 (0.5, 13.6) | **8.2** (1.6, 42.5) | 1.0 (0.4, 2.8) | **3.1** (1.1, 8.7) | 3.1 (1.0, 9.6) |
| DUD | 11 (26.8) | 16 (15.8) | 11 (19.0) | 5 (18.5) | 0.5 (0.2, 1.2) | 0.6 (0.2, 1.7) | 0.6 (0.2, 2.0) | 1.2 (0.5, 2.9) | 1.2 (0.4, 3.7) | 1.0 (0.3, 3.1) |

GAD: generalized anxiety disorder; SOP: social phobia; PD: panic disorder; AGO: agoraphobia; OCD: obsessive-compulsive disorder; PTSD: post-traumatic stress disorder; AUD: alcohol abuse/dependence disorder; DUD: drug abuse/dependence disorder

AOR: odd ratio adjusted for age and gender

Values in bold type indicated statistically significant.
