# Supplementary material for: Comparison of associated features and drug treatment between co-occurring unipolar and bipolar disorders in depressed eating disorder patients
Source: BMC Psychiatry. 2017 Feb 27;17:81. doi: 10.1186/s12888-017-1243-0 (PMC5327542; doi:10.1186/s12888-017-1243-0)
Supplement: Additional file 2: Table S 2.1. — Comparison of demographics and clinical variables for eating disorder patients (excluding AN) during major depressive episodes. Table S2.2. Comparison of psychological assessments and BMI among depressed eating disorder patients (excluding AN) with or without lifetime hypomania/mania episodes. Table S2.3. Comparison of lifetime psychiatric diagnoses among depressed eating disorder patients (excluding AN) with or without lifetime hypomania/mania episode. Table S2.4 Comparison of pharmacotherapy among depressed eating disorder patients (excluding AN) with or without lifetime hypomania/mania episode at enrollment. (DOCX 42 kb) [file 12888_2017_1243_MOESM2_ESM.docx]

Table S 2.1. Comparison of demographics and clinical variables for eating disorder patients (excluding AN) during major depressive episodes

| Variables | Eating disorders with  MDD (*n* = 101) | Eating disorders with  bipolar II disorder (*n* = 51) | Eating disorders with  bipolar I disorder (*n* = 34) | *P*-value |
| --- | --- | --- | --- | --- |
| Gender, female | 94 (93.1) | 42 (82.4) | 26 (76.5) | **0.0219** |
| Age (years) | 27.3 ± 7.1 | 28.2 ± 7.2 | 28.4 ± 8.5 | 0.6442 |
| Age at onset of disordered eating (years) | 20.3 ± 6.2 | 19.0 ± 4.3 | 20.4 ± 8.0 | 0.4362 |
| Age at onset of depression (years) | 23.0 ± 7.2 | 22.2 ± 6.8 | 21.4 ± 8.1 | 0.5649 |
| Education (years) | 14.4 ± 2.3 | 15.0 ± 2.4 | 13.2 ± 2.8 | **0.0029** |
| Eating disorder subtypes |  |  |  | 0.3512 |
| Bulimia Nervosa | 60 (59.4) | 25 (49.0) | 16 (47.1) |  |
| Binge eating Disorders | 25 (24.8) | 19 (37.3) | 14 (41.2) |  |
| Eating disorders NOS | 16 (15.8) | 7 (13.7) | 4 (11.8) |  |

MDD: major depressive disorder; AN: anorexia nervosa

Values indicate N (%) or Mean ± SD

Table S2.2 Comparison of psychological assessments and BMI among depressed eating disorder patients (excluding AN) with or without

lifetime hypomania/mania episodes

| Variables | Eating disorders with  MDD  (*n* = 101) (1) | Eating disorders with  bipolar II disorder  (*n* = 51) (2) | Eating disorders with  bipolar I disorder  (*n* = 34) (3) | 2 vs. 1  *P*-value^†^ | 3 vs. 1  *P*-value^†^ | 3 vs. 2  *P*-value^†^ |
| --- | --- | --- | --- | --- | --- | --- |
|  | Mean ± SD | Mean ± SD | Mean ± SD |  |  |  |
| Intelligence quotient | 100.4 ± 13.6 | 100.9 ± 12.4 | 91.6 ± 12.9 | 0.4170 | **0.0067** | **0.0019** |
| Working memory | 54.3 ± 26.2 | 55.3 ± 28.6 | 35.4 ± 27.1 | 0.7935 | **0.0005** | **0.0012** |
| Bulimic Investigatory Test Edinburgh | 31.0 ± 10.6 | 31.6 ± 9.3 | 29.0 ± 11.7 | 0.1794 | 0.8979 | 0.2723 |
| Symptom subscale | 21.3 ± 6.0 | 21.1 ± 5.5 | 18.9 ± 6.3 | 0.7207 | 0.0690 | 0.0769 |
| Severity subscale | 9.6 ± 6.2 | 10.5 ± 5.1 | 10.1 ± 7.1 | 0.0705 | 0.1956 | 0.9031 |
| Body Shape Questionnaire-8 | 4.8 ± 1.2 | 4.9 ± 1.0 | 4.4 ± 1.4 | 0.1294 | 0.4579 | 0.0507 |
| Beck Depression Inventory | 26.5 ± 10.6 | 27.1 ± 8.8 | 32.0 ± 10.0 | 0.8537 | **0.0207** | **0.0259** |
| Affective Lability Scale | 49.1 ± 8.8 | 51.6 ± 7.9 | 57.0 ± 8.9 | 0.0704 | **< 0.0001** | **0.0056** |
| Barrett Impulsiveness Scale | 58.3 ± 6.0 | 60.9 ± 6.5 | 63.0 ± 7.0 | **0.0235** | **< 0.0001** | 0.1382 |
| BMI, current (kg/m^2^) | 21.9 ± 4.2 | 23.9 ± 5.3 | 24.8 ± 5.5 | 0.0661 | **0.0429** | 0.6128 |
| BMI, maximal (kg/m^2^) | 24.1 ± 4.7 | 27.1 ± 6.4 | 26.6 ± 5.2 | **0.0093** | 0.1427 | 0.4833 |
| BMI, minimal (kg/m^2^) | 18.0 ± 2.6 | 19.1 ± 2.6 | 19.3 ± 3.4 | 0.1483 | 0.3441 | 0.9016 |

MDD: major depressive disorder; BMI: Body mass index; AN: anorexia nervosa

^†^ Adjusted for age, gender, and eating disorder subtypes.

Values in bold type indicated statistically significant.

Table S2.3 Comparison of lifetime psychiatric diagnoses among depressed eating disorder patients (excluding AN) with or without lifetime hypomania/mania

episode

| Variables | Eating disorders with  MDD  (*n* = 101) (1) | Eating disorders with  bipolar II disorder  (*n* = 51) (2) | Eating disorders with  bipolar I disorder  (*n* = 34) (3) | 2 vs. 1  AOR (95% CI) | 3 vs. 1  AOR (95% CI) | 3 vs. 2  AOR (95% CI) |
| --- | --- | --- | --- | --- | --- | --- |
|  | N (%) | N (%) | N (%) |  |  |  |
| Comorbid diagnosis |  |  |  |  |  |  |
| Generalized anxiety disorder | 43 (42.6) | 26 (51.0) | 19 (55.9) | 1.38 (0.69, 2.75) | 1.68 (0.75, 3.77) | 1.21 (0.49, 3.00) |
| Social phobia | 40 (39.6) | 19 (37.3) | 18 (52.9) | 0.96 (0.47, 1.96) | 1.90 (0.83, 4.36) | 1.98 (0.80, 4.87) |
| Panic disorder | 14 (13.9) | 14 (27.5) | 12 (35.3) | 2.22 (0.95, 5.19) | **3.09 (1.21, 7.88)** | 1.47 (0.56, 3.80) |
| Agoraphobia | 27 (26.7) | 12 (23.5) | 14 (41.2) | 0.81 (0.36, 1.79) | 1.81 (0.78, 4.20) | 2.48 (0.91, 6.75) |
| Obsessive compulsive disorder | 29 (28.7) | 18 (35.3) | 16 (47.1) | 1.32 (0.64, 2.75) | 2.12 (0.93, 4.83) | 1.61 (0.66, 3.93) |
| Post-traumatic stress disorder | 15 (14.9) | 11 (21.6) | 4 (11.8) | 1.70 (0.70, 4.11) | 0.83 (0.25, 2.77) | 0.47 (0.13, 1.65) |
| Alcohol abuse/dependence | 3 (3.0) | 12 (23.5) | 12 (35.3) | **12.86 (3.23, 51.15)** | **27.10 (6.39, 114.94)** | 2.05 (0.74, 5.70) |
| Drug abuse/dependence | 15 (14.9) | 9 (17.7) | 8 (23.5) | 1.18 (0.47, 2.96) | 1.66 (0.62, 4.49) | 1.41 (0.48, 4.20) |
| Functional impairment^a^ |  |  |  | 0.79 (0.39, 1.59) ^#^ | **4.58 (1.71, 12.29)** ^#^ | **5.88 (2.05, 16.86)** ^#^ |
| Severe and very severe | 50 (49.5) | 23 (45.1) | 28 (82.4) |  |  |  |
| Marked | 34 (33.7) | 18 (35.3) | 6 (17.7) |  |  |  |
| None and mild | 17 (16.8) | 10 (19.6) | 0 |  |  |  |
| Suicide acts^b^ | 21 (20.8) | 15 (29.4) | 13 (38.2) | 1.57 (0.71, 3.47) | 2.36 (0.99, 5.67) | 1.47 (0.58, 3.72) |
| Auto-aggressive behaviors^c^ | 35 (34.7) | 13 (25.5) | 15 (44.1) | 0.63 (0.29, 1.36) | 1.46 (0.64, 3.35) | 2.32 (0.89, 6.05) |

MDD: major depressive disorder; AN: anorexia nervosa

# Patients with severe and very severe impairments compared with all others.

Impairments and suicide were assessed using the Structured Interview on Anorexic and Bulimic Disorder, Expert-Assessment (SIAB-EX).

a: rated by the Item 53 ‘Was your work performance objectively impaired at work or in your household ?”. Severity of impairment is rated from 0 (=no) to 4 (=very severe); b: rated by the item 69 ‘Did you ever attempt to commit suicide?’; c: rated by the Item 70 ‘Did you ever hurt yourself intentionally?’. Severity of suicidal acts and auto-aggressive behaviors is rated from 0 (= symptom not present) to 4 (= symptom very much/very severely present) according to the number of attempts or seriousness of physical injury; here suicide acts indicated the severity more than one serious attempt or many minor attempts and auto-aggressive behaviors indicated the severity more than marked degree.

AOR: Odd ratio adjusting for age, gender, and eating disorder subtypes.

Values in bold type indicated statistically significant.

Table S2.4 Comparison of pharmacotherapy among depressed eating disorder patients (excluding AN) with or without lifetime hypomania/mania episode at

enrollment

| Variables | Eating disorders with  MDD  (*n* = 101) (1) | Eating disorders with  bipolar II disorder  (*n* = 51) (2) | Eating disorders with  bipolar I disorder  (*n* = 34) (3) | 2 vs. 1  AOR (95% CI) | 3 vs. 1  AOR (95% CI) | 3 vs. 2  AOR (95% CI) |
| --- | --- | --- | --- | --- | --- | --- |
|  | N (%) | N (%) | N (%) |  |  |  |
| Antidepressant monotherapy | 54 (54.6) | 30 (58.8) | 10 (31.3) | 1.25 (0.62, 2.56) | **0.38 (0.16, 0.93)** | **0.30 (0.11, 0.77)** |
| Mood stabilizer monotherapy | 0 | 1 (2.0) | 0 | NA | NA | NA |
| Atypical antipsychotic monotherapy | 18 (18.2) | 5 (9.8) | 8 (25.0) | 0.45 (0.15, 1.38) | 1.52 (0.54, 4.3) | 3.24 (0.91, 11.55) |
| Antidepressant plus mood stabilizers | 1 (1.0) | 0 | 1 (3.1) | NA | NA | NA |
| Antidepressant plus antipsychotic | 16 (16.2) | 10 (19.6) | 5 (15.6) | 1.25 (0.51, 3.06) | 0.90 (0.28, 2.82) | 0.74 (0.22, 2.55) |
| Antidepressant plus mood stabilizers and antipsychotics | 1 (1.0) | 1 (2.0) | 1 (3.1) | NA | NA | NA |
| Any antidepressant | 72 (72.7) | 41 (80.4) | 17 (53.1) | 1.69 (0.71, 4.04) | 0.42 (0.17, 1.02) | **0.24 (0.08, 0.67)** |
| Any mood stabilizer | 5 (5.1) | 3 (5.9) | 6 (18.8) | 1.10 (0.25, 4.88) | **3.95 (1.06, 14.71)** | 3.96 (0.90, 17.44) |
| Any atypical antipsychotic | 36 (36.4) | 16 (31.4) | 16 (50.0) | 0.77 (0.36, 1.62) | 1.74 (0.75, 4.08) | 2.26 (0.88, 5.78) |

MDD: major depressive disorder; AN: anorexia nervosa

Data mission for 2 and 2 persons in eating disorders with MDD and eating disorders with bipolar I disorder, respectively.

AOR: Odd ratio adjusted for age, gender, and eating disorder subtypes.

Values in bold type indicated statistically significant.
