# Supplementary material for: Comparison of associated features and drug treatment between co-occurring unipolar and bipolar disorders in depressed eating disorder patients
Source: BMC Psychiatry. 2017 Feb 27;17:81. doi: 10.1186/s12888-017-1243-0 (PMC5327542; doi:10.1186/s12888-017-1243-0)
Supplement: Additional file 3: Table S 3.1. — Comparison of lifetime psychiatric diagnoses among depressed anorexia nervosa patients with or without lifetime hypomania/mania episode. Table S 3.2. Comparison of lifetime psychiatric diagnoses among depressed bulimia nervosa patients with or without lifetime hypomania/mania episode. Table S 3.3. Comparison of lifetime psychiatric diagnoses among depressed binge eating disorder patients with or without lifetime hypomania/mania episode. (DOCX 39 kb) [file 12888_2017_1243_MOESM3_ESM.docx]

Table S 3.1. Comparison of lifetime psychiatric diagnoses among depressed anorexia nervosa patients with or without lifetime hypomania/mania episode

| Variables | AN  with  MDD  (1)  (*n* = 32) | AN  with bipolar II disorder  (2)  (*n* = 4) | AN  with bipolar I disorder  (3)  (*n* = 5) | 2 vs. 1 | 3 vs. 1 | 3 vs. 2 |
| --- | --- | --- | --- | --- | --- | --- |
|  | N (%) | N (%) | N (%) | AOR (95% CI) | AOR (95% CI) | AOR (95% CI) |
| Comorbid diagnosis |  |  |  |  |  |  |
| Generalized anxiety disorder | 7 (21.9) | 1 (25.0) | 1 (20.0) | NA | NA | 0.75 (0.03-17.51) |
| Social phobia | 6 (18.8) | 4 (100.0) | 0 (0.0) | NA | NA | NA |
| Panic disorder | 2 (6.3) | 0 (0.0) | 0 (0.0) | NA | NA | NA |
| Agoraphobia | 0 (0.0) | 1 (25.0) | 1 (20.0) | NA | NA | NA |
| Obsessive compulsive disorder | 9 (28.1) | 1 (25.0) | 1 (20.0) | NA | NA | 0.72 (0.03-17.81) |
| Post-traumatic stress disorder | 0 (0.0) | 0 (0.0) | 1 (20.0) | NA | NA | NA |
| Alcohol abuse/dependence | 2 (6.3) | 0 (0.0) | 0 (0.0) | NA | NA | NA |
| Drug abuse/dependence | 8 (25.0) | 1 (25.0) | 2 (40.0) | 1.04 (0.09-12.42) | 2.18 (0.28-17.26) | 2.06 (0.11-39.49) |
| Functional impairment^a^ |  |  |  | NA | NA | 1.39 (0.05-36.24) |
| Severe and very severe | 17 (53.1) | 3 (75.0) | 4 (80.0) |  |  |  |
| Marked | 11 (34.4) | 0 (0.0) | 1 (20.0) |  |  |  |
| None and mild | 4 (12.5) | 1 (25.0) | 0 (0.0) |  |  |  |
| Suicide acts^b^ | 2 (6.3) | 1 (25.0) | 1 (20.0) | NA | NA | 0.74 (0.03-17.55) |
| Auto-aggressive behaviors^c^ | 6 (18.8) | 2 (50.0) | 3 (60.0) | NA | NA | 1.53 (0.10-22.66) |

AN: anorexia nervosa; MDD: major depressive disorder.

# Patients with severe and very severe impairments compared with all others.

Impairments and suicide were assessed using the Structured Interview on Anorexic and Bulimic Disorder, Expert-Assessment (SIAB-EX).

a: rated by the Item 53 ‘Was your work performance objectively impaired at work or in your household ?”. Severity of impairment is rated from 0 (=no) to 4 (=very severe); b: rated by the item 69 ‘Did you ever attempt to commit suicide?’; c: rated by the Item 70 ‘Did you ever hurt yourself intentionally?’. Severity of suicidal acts and auto-aggressive behaviors is rated from 0 (= symptom not present) to 4 (= symptom very much/very severely present) according to the number of attempts or seriousness of physical injury; here suicide acts indicated the severity more than one serious attempt or many minor attempts and auto-aggressive behaviors indicated the severity more than marked degree.

AOR: odd ratio adjusted for age and gender.

Values in bold type indicated statistically significant.

Table S 3.2. Comparison of lifetime psychiatric diagnoses among depressed bulimia nervosa patients with or without lifetime hypomania/mania episode

| Variables | BN  with  MDD  (1)  (*n* = 60) | BN  with bipolar II disorder  (2)  (*n* = 25) | BN  with bipolar I disorder  (3)  (*n* = 16) | 2 vs. 1 | 3 vs. 1 | 3 vs. 2 |
| --- | --- | --- | --- | --- | --- | --- |
|  | N (%) | N (%) | N (%) | AOR (95% CI) | AOR (95% CI) | AOR (95% CI) |
| Comorbid diagnosis |  |  |  |  |  |  |
| Generalized anxiety disorder | 26 (43.3) | 10 (40.0) | 9 (56.3) | 1.02 (0.38-2.72) | 2.09 (0.64-6.81) | 2.21 (0.57-8.59) |
| Social phobia | 22 (36.7) | 11 (44.0) | 9 (56.3) | 1.41 (0.52-3.82) | 2.37 (0.71-7.89) | 1.66 (0.47-5.89) |
| Panic disorder | 7 (11.7) | 7 (28.0) | 5 (31.3) | 2.75 (0.79-9.54) | 2.92 (0.68-12.62) | 1.11 (0.26-4.80) |
| Agoraphobia | 18 (30.0) | 5 (20.0) | 5 (31.3) | 0.58 (0.18-1.84) | 1.06 (0.31-3.63) | 1.90 (0.42-8.60) |
| Obsessive compulsive disorder | 19 (31.7) | 8 (32.0) | 8 (50.0) | 0.95 (0.34-2.70) | 2.03 (0.63-6.54) | 2.12 (0.58-7.74) |
| Post-traumatic stress disorder | 11 (18.3) | 5 (20.0) | 1 (6.3) | 0.96 (0.28-3.35) | 0.25 (0.03-2.23) | 0.19 (0.02-2.30) |
| Alcohol abuse/dependence | 1 (1.7) | 5 (20.0) | 6 (37.5) | **16.33 (1.77-150.44)** | **41.88 (4.36-402.10)** | 2.58 (0.60-11.08) |
| Drug abuse/dependence | 8 (13.3) | 3 (12.0) | 5 (31.3) | 0.82 (0.19-3.52) | 2.68 (0.69-10.41) | 3.22 (0.59-17.51) |
| Functional impairment^a^ |  |  |  | 0.63 (0.23-1.74) | **15.84 (1.93-130.18)** | **29.55 (2.89-302.30)** |
| Severe and very severe | 28 (46.7) | 10 (40.0) | 15 (93.8) |  |  |  |
| Marked | 22 (36.7) | 12 (48.0) | 1 (6.3) |  |  |  |
| None and mild | 10 (16.7) | 3 (12.0) | 0 (0.0) |  |  |  |
| Suicide acts^b^ | 9 (15.0) | 8 (32.0) | 4 (25.0) | 2.72 (0.88-8.40) | 1.94 (0.49-7.58) | 0.68 (0.16-2.89) |
| Auto-aggressive behaviors^c^ | 21 (35.0) | 7 (28.0) | 8 (50.0) | 0.66 (0.23-1.90) | 1.68 (0.53-5.32) | 2.57 (0.69-9.60) |

BN: bulimia nervosa; MDD: major depressive disorder.

# Patients with severe and very severe impairments compared with all others.

Impairments and suicide were assessed using the Structured Interview on Anorexic and Bulimic Disorder, Expert-Assessment (SIAB-EX).

a: rated by the Item 53 ‘Was your work performance objectively impaired at work or in your household ?”. Severity of impairment is rated from 0 (=no) to 4 (=very severe); b: rated by the item 69 ‘Did you ever attempt to commit suicide?’; c: rated by the Item 70 ‘Did you ever hurt yourself intentionally?’. Severity of suicidal acts and auto-aggressive behaviors is rated from 0 (= symptom not present) to 4 (= symptom very much/very severely present) according to the number of attempts or seriousness of physical injury; here suicide acts indicated the severity more than one serious attempt or many minor attempts and auto-aggressive behaviors indicated the severity more than marked degree.

AOR: odd ratio adjusted for age and gender.

Values in bold type indicated statistically significant.

Table S 3.3 Comparison of lifetime psychiatric diagnoses among depressed binge eating disorder patients with or without lifetime hypomania/mania episode

| Variables | Binge eating disorders with MDD  (1)  (*n* = 25) | Binge eating disorders  with bipolar II disorder  (2)  (*n* = 19) | Binge eating disorders  with bipolar I disorder  (3)  (*n* = 14) | 2 vs. 1 | 3 vs. 1 | 3 vs. 2 |
| --- | --- | --- | --- | --- | --- | --- |
|  | N (%) | N (%) | N (%) | AOR (95% CI) | AOR (95% CI) | AOR (95% CI) |
| Comorbid diagnosis |  |  |  |  |  |  |
| Generalized anxiety disorder | 10 (40.0) | 14 (73.7) | 8 (57.1) | **4.65 (1.19-18.13)** | 1.73 (0.41-7.27) | 0.40 (0.08-1.89) |
| Social phobia | 8 (32.0) | 7 (36.8) | 5 (35.7) | 1.20 (0.34-4.24) | 1.06 (0.25-4.43) | 0.96 (0.22-4.30) |
| Panic disorder | 6 (24.0) | 4 (21.1) | 5 (35.7) | 0.86 (0.20-3.63) | 1.83 (0.41-8.07) | 2.26 (0.45-11.30) |
| Agoraphobia | 7 (28.0) | 3 (15.8) | 6 (42.9) | 0.44 (0.09-2.04) | 1.50 (0.35-6.38) | 3.91 (0.72-21.36) |
| Obsessive compulsive disorder | 4 (16.0) | 8 (42.1) | 7 (50.0) | 3.91 (0.93-16.44) | 4.62 (0.97-22.00) | 1.26 (0.30-5.34) |
| Post-traumatic stress disorder | 1 (4.0) | 6 (31.6) | 2 (14.3) | **12.52 (1.33-118.33)** | 5.37 (0.42-69.14) | 0.45 (0.07-2.86) |
| Alcohol abuse/dependence | 0 (0.0) | 4 (21.1) | 3 (21.4) | NA | NA | 1.10 (0.19-6.34) |
| Drug abuse/dependence | 5 (20.0) | 4 (21.1) | 2 (14.3) | 1.00 (0.22-4.48) | 0.53 (0.08-3.49) | 0.57 (0.08-3.90) |
| Functional impairment^a^ |  |  |  | 0.97 (0.29-3.24) | 2.49 (0.58-10.62) | 3.05 (0.66-14.05) |
| Severe and very severe | 12 (48.0) | 9 (46.4) | 10 (71.4) |  |  |  |
| Marked | 9 (36.0) | 4 (21.1) | 4 (28.6) |  |  |  |
| None and mild | 4 (16.0) | 6 (31.6) | 0 (0.0) |  |  |  |
| Suicide acts^b^ | 6 (24.0) | 5 (26.3) | 6 (42.9) | 1.02 (0.25-4.18) | 1.98 (0.45-8.70) | 2.15 (0.44-10.65) |
| Auto-aggressive behaviors^c^ | 7 (28.0) | 5 (26.3) | 6 (42.9) | 0.78 (0.19-3.22) | 1.52 (0.34-6.78) | 1.96 (0.37-10.41) |

MDD: major depressive disorder.

# Patients with severe and very severe impairments compared with all others.

Impairments and suicide were assessed using the Structured Interview on Anorexic and Bulimic Disorder, Expert-Assessment (SIAB-EX).

a: rated by the Item 53 ‘Was your work performance objectively impaired at work or in your household ?”. Severity of impairment is rated from 0 (=no) to 4 (=very severe); b: rated by the item 69 ‘Did you ever attempt to commit suicide?’; c: rated by the Item 70 ‘Did you ever hurt yourself intentionally?’. Severity of suicidal acts and auto-aggressive behaviors is rated from 0 (= symptom not present) to 4 (= symptom very much/very severely present) according to the number of attempts or seriousness of physical injury; here suicide acts indicated the severity more than one serious attempt or many minor attempts and auto-aggressive behaviors indicated the severity more than marked degree.

AOR: odd ratio adjusted for age and gender.

Values in bold type indicated statistically significant.
